# Fast reconstruction method of three-dimension model based on dual RGB-D cameras for peanut plant

**DOI:** 10.1186/s13007-023-00998-z

**Published:** 2023-02-27

**Authors:** Yadong Liu, Hongbo Yuan, Xin Zhao, Caihu Fan, Man Cheng

**Affiliations:** College of Mechanical and Electrical Engineering, Hebei Agricultural University, Baoding, 071001 China

**Keywords:** Peanut plant, Three-dimensional model, Point cloud, Coordinate transformation, Kinect v2

## Abstract

**Background:**

Plant shape and structure are important factors in peanut breeding research. Constructing a three-dimension (3D) model can provide an effective digital tool for comprehensive and quantitative analysis of peanut plant structure. Fast and accurate are always the goals of the plant 3D model reconstruction research.

**Results:**

We proposed a 3D reconstruction method based on dual RGB-D cameras for the peanut plant 3D model quickly and accurately. The two Kinect v2 were mirror symmetry placed on both sides of the peanut plant, and the point cloud data obtained were filtered twice to remove noise interference. After rotation and translation based on the corresponding geometric relationship, the point cloud acquired by the two Kinect v2 was converted to the same coordinate system and spliced into the 3D structure of the peanut plant. The experiment was conducted at various growth stages based on twenty potted peanuts. The plant traits’ height, width, length, and volume were calculated through the reconstructed 3D models, and manual measurement was also carried out during the experiment processing. The accuracy of the 3D model was evaluated through a synthetic coefficient, which was generated by calculating the average accuracy of the four traits. The test result showed that the average accuracy of the reconstructed peanut plant 3D model by this method is 93.42%. A comparative experiment with the iterative closest point (ICP) algorithm, a widely used 3D modeling algorithm, was additionally implemented to test the rapidity of this method. The test result shows that the proposed method is 2.54 times faster with approximated accuracy compared to the ICP method.

**Conclusions:**

The reconstruction method for the 3D model of the peanut plant described in this paper is capable of rapidly and accurately establishing a 3D model of the peanut plant while also meeting the modeling requirements for other species' breeding processes. This study offers a potential tool to further explore the 3D model for improving traits and agronomic qualities of plants.

## Background

Peanuts are a widely cultivated oil and cash crop, providing a significant source of oil and protein [1]. The global total peanut production was 50.606 million tons, and China was the largest producer with 18.20 million tons in 2021 [2]. It is important to improve the yield and quality of peanuts for China’s and the world’s oil supply [3, 4]. An effective way to increase peanuts production is by developing new varieties with excellent traits using advanced gene technology [5–7]. The results of interaction between genotypes and environmental factors are expressed through the phenotypic parameters of plant structure [8, 9]. Plant architectural traits are important phenotypic traits for selecting new adaptative cultivars in crop breeding studies [10].

Plant structure reflects the size and organization form of above-ground organs of crops [11], which can indicate growth status, cultivation conditions, and water and fertilizer measures of crops [12]. In addition, the phenotypic traits of plants such as height and width also provide references for breeders to cultivate excellent breeding [13–16]. The establishment of a three-dimensional (3D) model of the plant can comprehensively understand the morphological features of the plant, which avoid the limitation of two-dimensional (2D) imaging lacking depth information and facilitate the subsequent accurate extraction of multiple trait parameters [10, 17–19]. Therefore, the 3D reconstruction model of plants has gradually become an essential part of phenotypic research.

In the last decade, a lot of research has been done on the 3D modeling of plant structures using different kinds of technologies including stereo vision (SV), Structure from Motion (SfM), LiDAR, and RGB-D camera. Both SV and SfM 3D modeling methods are based on 2D imaging devices, which reconstruct the 3D architecture of the target by 2D images from different perspectives. SV uses two or more cameras to collect the images of the target at the same time, while SfM captures overlapping images by moving the camera around an object [20–22]. Bao et al. [23] used a stereovision-equipped robot to reconstruct a 3D model of sorghum and successfully acquired phenotypic data from high-throughput crops in the field. Malambo et al. [24] proposed a 3D modeling method for SfM based on an unmanned aerial vehicle system and estimated maize and sorghum height data from point clouds generated using SfM. SV and SfM can be used both indoors and field, and in SfM the camera can be mounted on the unmanned aerial vehicle (UAV) platform to quickly obtain information on a large area of the field in a short time [25]. However, SV and SfM are sensitive to light intensity, and the change in light environment will increase the deviation of the image. Although the image quality requirement can be reduced in the SfM method, there is a lot of data redundancy in multiple images and the reconstruction speed is slow [26]. It should be concerned and considered that improve the speed of the 3D modeling. In addition, some researchers try to restore depth information through deep learning algorithms based on RGB images [27–29]. However, this technology requires high-quality RGB images and powerful computers to implement and has not been widely used in plant 3D modeling.

It is a widely used technique for reconstructing a 3D canopy using scanning equipment to generate a 3D point cloud of plants, which generally employs the time of flight (ToF) or phase-shifting scanning principle to generate the point cloud [30]. Shi et al. [31] used LiDAR to create 3D models of corn plants and enable real-time monitoring of crops' 3D information. Moreno et al. [32] reconstructed vineyard crops through 3D points cloud generated by LiDAR installed onboard a mobile platform. Leaf Color is a key phenotype trait of crops, and the 3D model with color information can provide more phenotype information for simulating dynamic crop growth and development in space and time [33]. As an active 3D imaging instrument, the LiDAR is more costly than the 2D camera [34]. Moreover, the reconstruction effect is affected by the edge effect, the diffuse reflection occurs when the excited wave is projected to the branch or leaf's edge, then the lidar may miss the reflected wave, impairing edge recognition [35, 36]. The RGB-D camera can acquire both color and depth information about the target simultaneously. Its advantages include ease of development, high real-time performance, and strong anti-interference properties [37, 38]. Thus, since Microsoft released the Kinect in 2010, an increasing number of researchers have applied it to plant 3D modeling [39–42].

The 3D modeling methods have been applied in the field environment, and the plant phenotypic traits of crop populations are obtained through the 3D reconstruction model of crops [43]. Although the field experiment can reflect the performance of crops in the actual growth environment, it is easy to be affected by many uncontrollable factors, such as weather, light intensity, and wind [26, 44]. Moreover, breeding programs require the evaluation of architectural traits at a finer scale, such as organ scale [10, 45, 46]. However, it is not a feasible measurement of those traits under the field conditions, so the researchers tend to conduct the initial screening of breeds in a controlled environment because the changes in the process of crop growth can be found more intuitively [17, 47, 48]. As an RGB-D camera, Kinect v2 shows great potential with low cost and strong robustness in 3D modeling indoors [21, 49], and it has been applied to 3D fine modeling of plants [30, 50–52].

Fusing point clouds obtained from multiple angles is a common method to establish accurate 3D models, and researchers tend to reconstruct plant modeling through three or more angle point clouds data [42, 49]. The premise of point cloud fusion is to realize the registration of multiple point clouds, which accurately align the point cloud data from different views into the complete 3D model of the plant [17]. The registration algorithm can find the relationship between different views by searching the correspondence of key points between multiple views. The accuracy of 3D modeling is determined by the registration algorithm, as a classical 3D point cloud registration algorithm, the iterative closest point (ICP) algorithm has been widely used in plant modeling [38, 53]. It is difficult to establish an accurate plant 3D model based on the information collected from one view, so it is necessary to scan the target from multiple views to obtain point clouds in different directions and integrate them effectively [54]. Point clouds from more perspectives will improve the accuracy of modeling, but the more point clouds for registration, the longer the time required to establish the model, and the modeling efficiency can decrease as the number of points increases [55]. The relationship between balancing speed and accuracy is a problem to be considered.

At present, many achievements have been made in the reconstruction of the 3D model such as Maize [31], Sorghum [23], and Soybean [56], but the 3D plant model of the peanut has not been thoroughly researched. This research aims to quickly establish an accurate 3D model of an individual peanut plant by point clouds obtained from only two views. This paper describes this 3D modeling method, which can quickly reconstruct the 3D model through the non-overlapping point clouds in two symmetrical directions and can ensure the accuracy of the 3D model. The main contributions of this paper are on the following aspects: (1) proposing a method for automatic 3D plant reconstruction and phenotypic data acquisition for peanuts based on dual Kinect v2. (2) optimizing the parameters of the filtering algorithm, and evaluating the accuracy of the reconstructed 3D point cloud model to determine the method's feasibility. (3) designing a comparison experiment with the ICP algorithm to test the rapidity of the method proposed in this paper.

This paper is organized as follows: "Related works" section explained the related works, which include the point cloud acquisition system of the peanut plant, parameter calibration of Kinect v2, and generation of the color 3D point cloud. "Methods" section describes the method for 3D plant reconstruction and phenotypic data acquisition for the peanut plant. "Experiment and results" section reports the experiment setup and results to determine the effectiveness of the method proposed in this paper. "Disscussion" section discusses the factors affecting the accuracy of the 3D model reconstruction, the importance of parameter selection in statistical filtering, and the advantages of the method proposed in this paper in modeling speed. Finally, the conclusions and future work are given in "Conclusion" section.

## Related works

In this section, the acquisition method of peanut plant point cloud is introduced, which include the acquisition system of point cloud, sensor calibration and the generation process of 3D point cloud.

### Peanut plant point cloud acquisition system

**Fig. 1:**
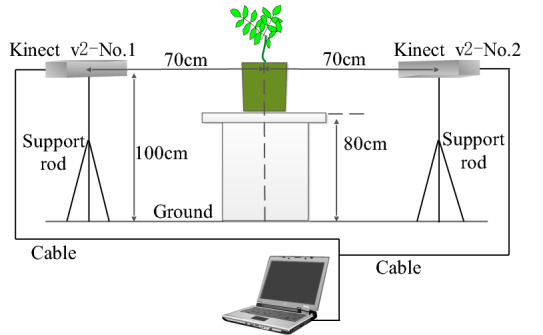
Schematic diagram of point cloud data acquisition system

To collect the peanut plant point cloud, a plant information acquisition system based on Kinect v2 was developed (Fig. 1). The flowerpot, which measures 23 cm in diameter and 20 cm in height, was placed on an 80 cm-high operating table when data collection was implemented. Two Kinect v2, designated No.1 and No.2, were placed on opposite sides of the flowerpot with left and right mirror symmetry to reduce the effect caused by blade overlap. Normally, the height and width of peanut plants do not exceed 40 cm [13, 57]. The measurement range of Kinect v2 is from 50 to 400 cm, and the Kinect v2 depth camera has a vertical field of view of 60 degrees [58]. Two Kinect v2 were 70 cm away from the center of the flowerpot and the lens’s focal point was 100 cm above the ground, which can meet the measurement requirements and fill the viewing field with peanut plants to the greatest extent.

The resolution of color and depth images got by Kinect v2 is 1920×1080 and 512×424, respectively. The image information obtained by Kinect v2 is transferred to the computer. The CPU is Intel(R) Core (TM) I5-7300HQ CPU @ 2.50 GHz, the graphics card is NVIDIA GEFORCE GTX1050 Ti, and the operating system is Microsoft Windows 10. A 3D reconstruction model program is developed based on C +  + , OpenCV3.4.1, and PCL (point cloud library) 1.8.1.

### Kinect v2 parameter calibration

To obtain accurate color and depth information of a targeted object, the RGB-D camera needs to be carefully calibrated to achieve pixel-to-pixel matching between its depth image and RGB image. The calibration is affected by the RGB-D camera’s intrinsic parameters, e.g. focal length, lens distortion, and the relative position and orientation between RGB and depth sensors. These parameters differ from camera to camera and should be provided by their manufacturer. However, some commercial RGB-D cameras do not come with detailed technical information [59] and the point clouds which form the depth images are often very noisy which makes numerous challenges to use RGB-D cameras correctly and accurately. To more accurately align RGB images and depth images and create color 3D point clouds, Kinect v2 was calibrated for intrinsic parameters.

**Fig. 2:**
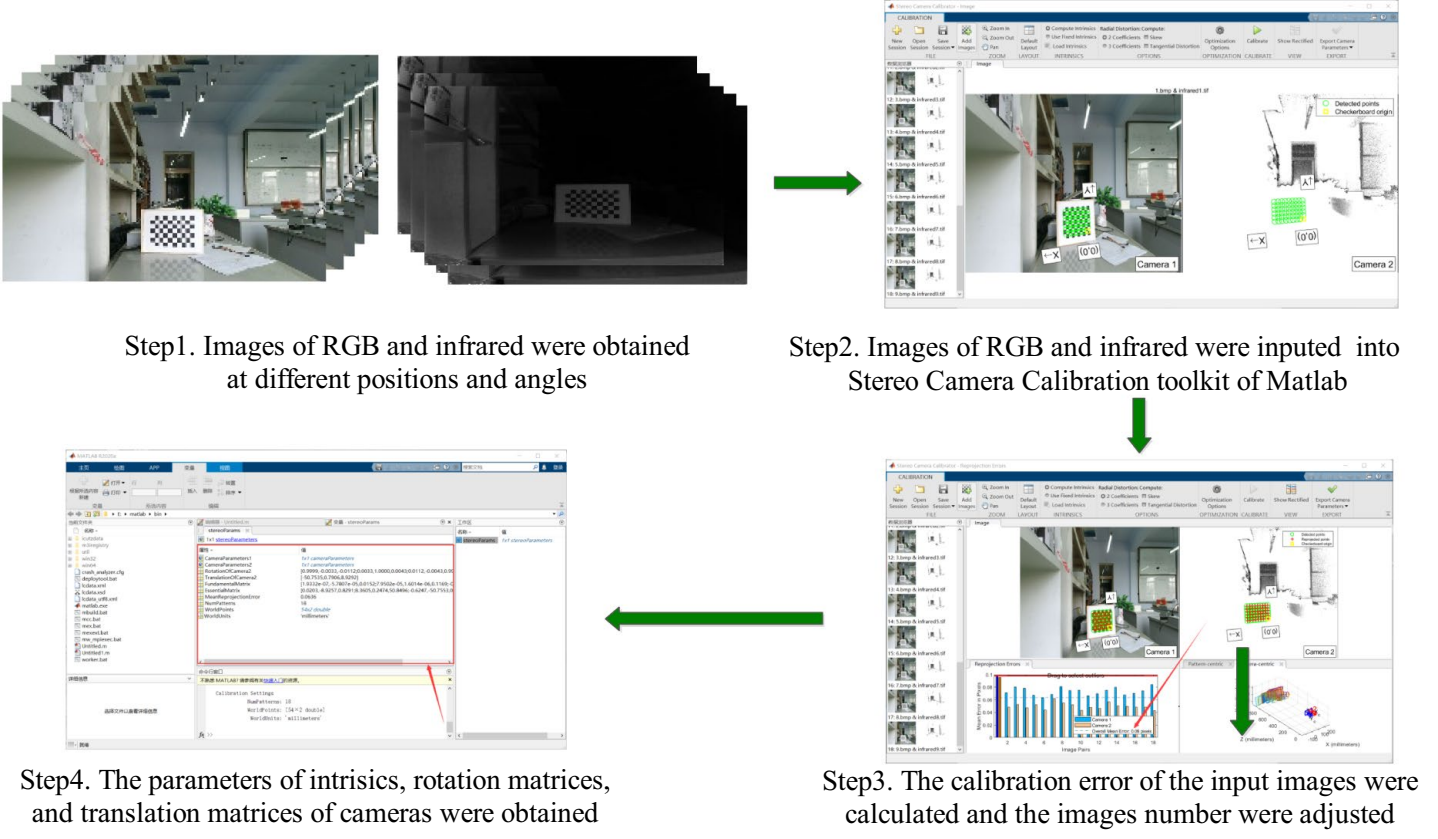
Calibration of Kinect v2 camera parameters

The Kinect v2 is equipped with an RGB camera and a depth camera that does not overlap. To obtain the intrinsic parameter matrices ${IM}_{rgb}$ and ${IM}_{ir}$ for the RGB and depth cameras, respectively, as well as the rotation matrix ${EM}_{r}$ and translation matrix ${EM}_{t}$ between the RGB and depth cameras, the Kinect v2 parameters must be calibrated to match color and depth images. RGB and depth images can be matched using these matrices. Figure 2 illustrates the steps involved in calibrating the Kinect v2’s parameters [60].

Step 1: Kinect v2 was used to take color and infrared images of 20 checkerboard calibration plates at different positions, angles, and attitudes.

Step 2: The captured images were input into the Stereo Camera Calibration software package of MATLAB.

Step 3: The stereo camera calibration software package was used to determine the calibration error of the input image. The image with the largest calibration error will then be deleted in descending order until the average calibration error is less than 0.15.

Step 4: The intrinsic parameter matrices ${IM}_{rgb}$ and ${IM}_{ir}$, rotation matrices ${EM}_{r}$, and translation matrices ${EM}_{t}$ of RGB camera and depth camera were got, respectively.

### Generation process of color 3D point cloud

The depth data obtained by Kinect v2 represents the distance between the target point and the plane where Kinect v2 was located. The 3D reconstruction of the target was based on the coordinate information of the target point, so the depth data should be converted into a 3D point cloud containing coordinate information, as shown in Eq. (1).

1 $$ \left\{ \begin{gathered} p_{ir} \, = \,\,d \times \,[x^{*} \,\,y^{*} \,\,1] \hfill \\ P_{ir} \, = \,P_{ir} \times \,IM_{ir} \hfill \\ \end{gathered} \right.\, $$

where ${p}_{ir}$ represents the depth information of the pixel in the depth image got by Kinect v2, $d$ represents the depth value, ${x}^{*}$, ${y}^{*}$ distribution represents the row and column positions of the pixel in the depth image. ${P}_{ir}$ represents the transformed point cloud information.

To get the color point cloud, the depth point cloud from the depth image needs to be converted to the RGB camera coordinate system. This transformation can be obtained based on the calibrated rotation ${EM}_{r}$ and translational matrix ${EM}_{t}$ by using Eq. (2).

2 $${P}_{rgb}={P}_{ir}\times {EM}_{r}+{EM}_{t} $$

where ${P}_{rgb}$ represents any point in the depth point cloud converted to the color camera coordinate system. Since the imaging range and resolution of the depth image and color image are different, it is necessary to find the corresponding point of ${P}_{rgb}$ point in the color image to get its color information. The pixels $CP$ matching ${P}_{rgb}$ positions in color images can be obtained by Eq. (3).

3 $$CP={P}_{rgb}\times {IM}_{rgb}$$

The $CP$ consists of vectors $\left(\widehat{x},\widehat{y},c\right)$, $\widehat{x}$ and $\widehat{y}$ are the computed values of the row and column positions of the color image. The color information ‘*c*’ of the points closest to these two calculated values in the actual color image is considered the color value of the corresponding depth point cloud. Based on this method, color three-dimensional point clouds can be obtained.

## Methods

In the following section, the method of fast 3D model reconstruction was presented. Firstly, the point cloud data was filtered twice, and then two pieces of the point cloud from dual Kinect v2 were fusion and modeled based on the corresponding position relationship in space. The evaluation method used for the accuracy of the 3D reconstruction model has also been mentioned in this section.

### Passthrough filtering and parameter determination

**Fig. 3:**
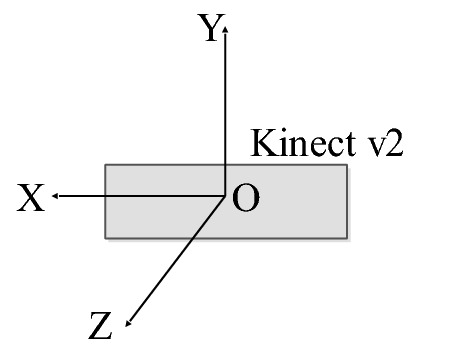
The coordinate system of Kinect v2

When the RGB-D camera acquires depth point cloud information, background noise is introduced, which can be removed using PassThrough filtering [61]. The PassThrough filtering can eliminate points that do not satisfy the constraint conditions, as illustrated in Eq. (4), then the region of interest (ROI) can be obtained.

4 $$\left\{\begin{array}{c}{X}_{min}<x<{X}_{max}\\ {Y}_{min}<y<{Y}_{max}\\ {Z}_{min}<z<{Z}_{max}\end{array}\right.$$

where $x$, $y$, and $z$ denote the coordinate system position of the point cloud, $\left({X}_{min},{X}_{max}\right)$, $\left({Y}_{min},{Y}_{max}\right)$, and $\left({Z}_{min},{Z}_{max}\right)$ denote the filtering range in the coordinate system's three coordinate directions (Fig. 3). Their values can be calculated based on the size of the peanut plant, which were shown in Table1. All point clouds that do not satisfy this constraint condition were filtered out as background noise, and the ROI was determined following PassThrough filtering.

**Table 1:** Calibration results of Kinect v2 parameters

Parameters	Value	Explain
${X}_{min}$	− 20 cm	Peanut was planted in the center of the flowerpot, and the Kinect v2’s lens was aligned with the center of the flowerpot. Normally, the width of peanut plants does not exceed 40 cm
${X}_{max}$	20 cm	
${Y}_{min}$	0 cm	Kinect v2's lens was at the same height as the upper edge of the flowerpot. Normally, the height of peanut plants does not exceed 40 cm
${Y}_{max}$	40 cm	
${Z}_{min}$	50 cm	The data measured by Kinect v2 in the Z-axis direction is the distance from the target to the lens. Kinect v2 was 70 cm away from the center of the flowerpot, and the width of the peanut plants is no more than 40 cm
${Z}_{max}$	90 cm	

### Statistical filtering and parameter optimization

After PassThrough filtering, only the approximate existence range of an effective point cloud can be obtained. There are still some noises in these point clouds caused by the environment and the camera, which must be removed via the second filtering. The second filtering method is statistical filtering [61], which is based on the assumption that the average distance between all points in the point cloud and their m neighboring points follows the Gaussian distribution. During statistical filtering, Eq. (5) is used to calculate the average distance between each point in the point cloud and its $m$ neighboring points. After that, determine the mean value $\mu$ and standard deviation $\sigma$ of the mean distance between each point in the point cloud. Finally, the effective point cloud range is determined as $\left(\mu -k \cdot \sigma ,\mu +k \cdot \sigma \right)$, and $k$ is the coefficient. If the average distance between a point in the point cloud and m neighboring points is not within this range, the point is considered to be noise. The values of $m$ and $k$ in statistical filtering influence the filtering effect.

5 $$\overline{d }=\frac{1}{m}\sum_{j=1}^{m}\sqrt{{\left({x}_{i}-{x}_{j}\right)}^{2}+{\left({y}_{i}-{y}_{j}\right)}^{2}+{\left({z}_{i}-{z}_{j}\right)}^{2}}$$

where ${x}_{i}$, ${y}_{i}$, and ${z}_{i}$ respectively represent the coordinate values of the target point on the three coordinate axes, ${x}_{j}$, ${y}_{j}$, and ${z}_{j}$ respectively represent the coordinate values of the nearest points of the target point on the three coordinate axes.

The value of $m$ is related to the number of point clouds of the target object, that is, the value of $m$ is affected by the point cloud density. The value of $k$ is related to $m$. The smaller the value of $m$, the less the number of point clouds output by statistical filtering under the condition of a constant $k$ value. The speed of the modeling will be improved by reducing the number of point clouds on the premise that the three-dimensional structure of the peanut plant is not distorted. An algorithm is designed to optimize the $k$ and $m$ values for improving the filtering effect, that is, selecting different $k$ and $m$ values to change the number of output 3D point clouds and spatial 3D structure, then determining the optimized parameter values by comparing the output effect. The steps of the algorithm are shown in the following.

Step 1: The value of $k$ is set to 1, and the $m$ value gradually decreases from 100 until the filtered point cloud spatial 3D structure starts to deteriorate significantly. The $m$ value before this phenomenon is considered the appropriate $m$ value.

Step 2: The value of $m$ is set to the value determined in step 1, and the $k$ value gradually increases from 0 until the filtered point cloud spatial 3D structure starts to deteriorate significantly. The $k$ value before this phenomenon is considered the appropriate $k$ value.

Step 3: The values of $m$ and $k$ obtained in the above steps are applied to the statistical filtering process as optimized parameters.

After optimizing the parameters, the number of point clouds after statistical filtering balances the accuracy and speed in the subsequent modeling process.

### Fusion and modeling of point clouds of peanut plant

The filtered point clouds can be directly fused to generate a 3D model if the exact coordinates of each point in the point clouds obtained by two Kinects can be determined in the same spatial coordinate system. The position information of the point cloud acquired by Kinect v2 is determined by the coordinate system in which it is located. In the real world, the same point has a different coordinate position in each of the Kinect v2 coordinate systems. The coordinate system of the Kinect v2 in various positions must be converted to the same coordinate system in order to restore the point cloud relative positions in the real world [62].

**Fig. 4:**
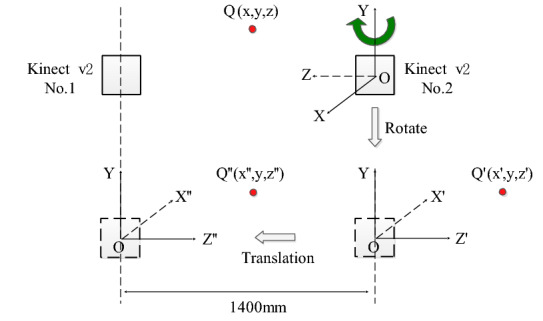
Transformation diagram of the coordinate system

In this paper, a 3D model reconstruction method based on point cloud spatial coordinate (PSC) is designed. To determine the spatial coordinate position of the point cloud, the coordinate system where Kinect v2-No.1 is used as the reference coordinate system, and the coordinate system of Kinect v2-No.2 is converted to Kinect v2-No.1. The conversion method is shown in Fig. 4, and the fusion and modeling steps of point clouds in the PSC method are shown in the following.

Step 1: Kinect v2-No.2 keeps the Y-axis unchanged and rotates 180 degrees to the right, then the position of $Q(x,y,z)$ in the original coordinate system is changed to $Q^{\prime}\,(x^{\prime},\,y^{\prime},\,z^{\prime})$ in the rotated coordinate system. The relation between *Q′* and *Q* is shown in Eq. (6).

6 $$\left\{\begin{array}{c}{x}^{^{\prime}}=-x\\ {y}^{^{\prime}}=y\\ {z}^{^{\prime}}=-z\end{array}\right.$$

Step 2: The rotating coordinate system of Kinect v2-No.2 moves 1400 mm to the left along the Z-axis, then the position of point $Q^{\prime}\,(x^{\prime},\,y^{\prime},\,z^{\prime})$ changes to $Q"(x", y", z")$. The relation between *Q"* and *Q*′ is shown in Eq. (7).

7 $$ \left\{ \begin{gathered} x^{\prime\prime}\, = \,x^{\prime} \hfill \\ y^{\prime\prime}\, = \,y^{\prime} \hfill \\ z^{\prime\prime}\, = \,z^{\prime}\, - \,1400 \hfill \\ \end{gathered} \right.\, $$

Step 3: The color 3D point cloud coordinates from Kinect v2-No.2 can be converted to the coordinate system in which Kinect v2-No.1 is located using the method described as follows:

8 $$\left[{x}^{\prime\prime},{y}^{\prime\prime},z^{\prime\prime}\right]=\left[x,y,z\right]\left[\begin{array}{ccc}-1& 0& 0\\ 0& 1& 0\\ 0& 0& -1\end{array}\right]-\left[\begin{array}{ccc}0& 0& 1400\end{array}\right]$$

Step 4: The color point clouds originating from Kinect v2-No.1 and Kinect v2-No.2 are spliced directly according to the transformed coordinate position, and then the 3D model of the peanut plant is generated.

### Accuracy evaluation of 3D reconstruction model of peanut plant

A common method of evaluating the accuracy of the 3D reconstruction model is to compare the phenotypic parameters calculated from the 3D model with those measured manually. These parameters are generally height, width, and volume [20, 50]. Based on the reconstructed 3D model, the height, width, length, and volume of the peanut plant were calculated through Eq. (9).

9 $$\left\{\begin{array}{c}{H}_{c}={Y}_{h\_max}-{Y}_{h\_min}\\ \begin{array}{c}{W}_{c}={X}_{w\_max}-{X}_{w\_min}\\ \begin{array}{c}{L}_{c}={Z}_{l\_max}-{Z}_{l\_min}\\ {V}_{c}={H}_{c}\times {W}_{c}\times {L}_{c}\end{array}\end{array}\end{array}\right.$$

where, the ${H}_{c}$, ${W}_{c}$, ${L}_{c}$, and ${V}_{c}$ respectively represent the height, width, length, and volume of the 3D model of the peanut plant. ${Y}_{h\_max}$_x_, ${X}_{w\_max}$, and ${Z}_{l\_max}$ represent the maximum value of the 3D model on the three coordinate axes respectively, and ${Y}_{h\_min}$, ${X}_{w\_min}$ and ${Z}_{l\_min}$ represent the minimum value of the 3D model on the three coordinate axes respectively.

The ground-truth data were obtained by taking manual measurements. The parameters of the peanut plant were measured with a ruler, and each of them was measured three times and the average value was taken. The synthetic accuracy of 3D model reconstruction was evaluated through Eq. (10).

10 $$Acc=\left[1-\left(\left|\frac{{H}_{c}-{H}_{m}}{{H}_{m}}\right|\times \frac{1}{4}+\left|\frac{{W}_{c}-{W}_{m}}{{W}_{m}}\right|\times \frac{1}{4}+\left|\frac{{L}_{c}-{L}_{m}}{{L}_{m}}\right|\times \frac{1}{4}+\left|\frac{{V}_{c}-{V}_{m}}{{V}_{m}}\right|\times \frac{1}{4}\right)\right]\times 100\%$$

where $Acc$ represents the model accuracy, and ${H}_{m}$, ${W}_{m}$, and ${L}_{m}$ respectively represent the actual measurement results. The ${V}_{m}$ was the volume of the peanut plant calculated from the actual measured values.

## Experiment and results

In this section, we presented the experimental design and results of the 3D model reconstruction of the peanut plant.

### Environment and scheme design of experiment

The experiment was carried out in the greenhouse of Hebei Agricultural University (115°28′35 "E, 38°50′57" N) from May 2021 to July 2021, and peanut variety Jihua No. 5 was planted. The planting method was potted, and the planting time was May 19, 2021. Twenty peanut plants with good growth were randomly chosen for the experiment. The experiment collected data on three distinct stages of peanut growth: sprout, seedling, and flowering stage, with collection dates of June 8, June 18, and July 1, respectively. For each peanut plant, two Kinect v2 captured one frame respectively when each experiment, and generated a group of point clouds. A group of point clouds of each peanut plant was acquired once in each grown stage, and three groups of point clouds were collected from each peanut plant during the whole experiment. A total of 60 groups of point clouds were obtained for twenty peanut plants throughout the whole experiment.

### Data filtering results

**Fig. 5:**
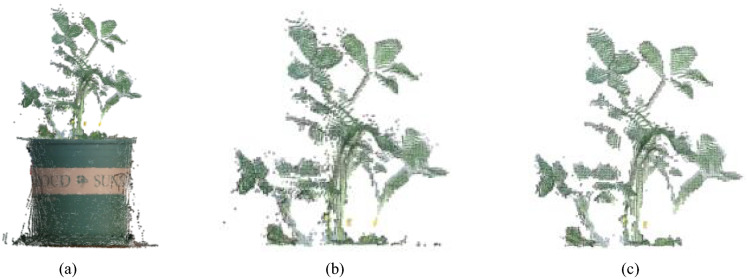
Data filter results. **a** 3D color point cloud. **b** PassThrough filtering result. **c** Statistical filtering result

Figure 5 illustrates data filtering results on the original 3D point cloud. Figure 5a shows the original 3D color point cloud before filtering, which is obtained by a side view Kinect v2. The effect of PassThrough filtering is depicted in Fig. 5b. After PassThrough filtering, only the point cloud containing the peanut plant is retained. The effect of statistical filtering is depicted in Fig. 5c. Certain interference and outlier noises are eliminated following statistical filtering. Compared to the effect of straight-through filtering, statistical filtering connects most point clouds, clears discrete point clouds, and clarifies the edges of the peanut plant.

### Results of 3D model construction

**Fig. 6:**
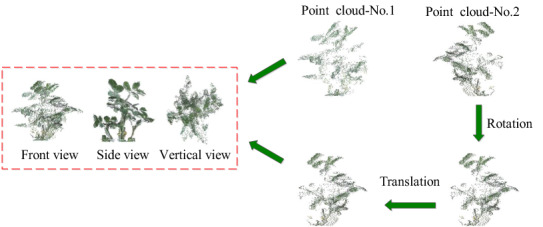
3D Reconstruction process and results

Figure 6 illustrates the process and results of reconstructing the peanut plant model using the filtered point cloud. As illustrated in the figure, two Kinect v2 point clouds splice together following conversion to form a complete three-dimensional peanut plant structure. Figure 6 shows that the point cloud density at the edge of the peanut plant 3D model is low due to the dual effects of filtering and diffuse reflection. The point cloud density in the center of the model is high, where the point cloud obtained by two Kinect v2 are overlapping.

### Accuracy evaluation result of 3D model

The experimental data were collected at three distinct stages of peanut growth, and during each experiment, twenty targets were reconstructed in three dimensions. The statistical data of ground- truth and calculated values based on the 3D model for geometric traits of peanut plants at the sprout stage are shown in Table 2. As shown in Table 2, the maximum accuracy of the calculated value of the peanut plant’s height relative to the real value on the ground-truth is 99.37%, and the minimum is 91.06%. The maximum accuracy of the calculated value of the peanut plant’s width relative to the real value on the ground-truth is 100.00%, and the minimum is 82.33%. The maximum accuracy of the calculated value of the peanut plant’s length relative to the real value on the ground-truth is 99.05%, and the minimum is 69.63%. The maximum accuracy of the calculated value of the peanut plant’s space volume relative to the real value on the ground-truth is 99.74%, and the minimum is 73.12%.

**Table 2:** Statistical data of geometric traits obtained by measured and 3D model calculated of peanut plant at sprout stage

Peanut plant	Height (mm)			Width (mm)			Length (mm)			Volume (cm^3^)		
	$M$	$C$	$AP$ (%)	$M$	$C$	$AP$ (%)	$M$	$C$	$AP$ (%)	$M$	$C$	$AP$ (%)
1	102	93	91.18	176	175	99.43	101	124	77.23	1813.15	2018.10	88.70
2	134	137	97.76	103	108	95.15	115	110	95.65	1587.23	1627.56	97.46
3	157	153	97.45	129	126	97.67	115	113	98.26	2329.10	2178.41	93.53
4	159	160	99.37	80	82	97.50	92	98	93.48	1170.24	1285.76	90.13
5	148	149	99.32	179	182	98.32	120	110	91.67	3179.04	2982.98	93.83
6	159	152	95.60	167	167	100.00	210	212	99.05	5576.13	5381.41	96.51
7	135	138	97.78	126	136	92.06	117	124	94.02	1990.17	2327.23	83.06
8	110	105	95.45	118	118	100.00	141	128	90.78	1830.18	1585.92	86.65
9	155	151	97.42	190	171	90.00	141	157	88.65	4152.45	4053.90	97.63
10	90	88	97.78	215	214	99.53	135	176	69.63	2612.25	3314.43	73.12
11	124	116	93.55	232	273	82.33	181	164	90.61	5207.01	5193.55	99.74
12	126	133	94.44	137	139	98.54	156	161	96.79	2692.87	2976.41	89.47
13	158	150	94.94	219	213	97.26	162	157	96.91	5605.52	5016.15	89.49
14	65	69	93.85	147	144	97.96	138	103	74.64	1318.59	1023.41	77.61
15	134	129	96.27	150	126	84.00	144	177	77.08	2894.40	2876.96	99.40
16	123	112	91.06	214	209	97.66	183	171	93.44	4816.93	4002.77	83.10
17	165	156	94.55	196	202	96.94	139	147	94.24	4495.26	4632.26	96.95
18	124	128	96.77	168	148	88.10	146	131	89.73	3041.47	2481.66	81.59
19	125	121	96.80	146	139	95.21	158	143	90.51	2883.50	2405.12	83.41
20	162	168	96.30	295	282	95.59	156	129	82.69	7455.24	6111.50	81.98

The $M$ represents the measured values, the $C$ represents calculated values based on the 3D model. The $AP$ represents the accuracy percentage of calculated values, and $AP=(1-|M-C|/M)100\%$

The statistical data of ground- truth and calculated values based on the 3D model for geometric traits of peanut plants at the seedling stage are shown in Table 3. As shown in Table 3, the maximum accuracy of the calculated value of the peanut plant’s height relative to the real value on the ground-truth is 99.64%, and the minimum is 95.78%. The maximum accuracy of the calculated value of the peanut plant’s width relative to the real value on the ground-truth is 99.10%, and the minimum is 85.15%. The maximum accuracy of the calculated value of the peanut plant’s length relative to the real value on the ground-truth is 99.66%, and the minimum is 80.33%. The maximum accuracy of the calculated value of the peanut plant’s space volume relative to the real value on the ground-truth is 96.70%, and the minimum is 75.70%.

**Table 3:** Statistical data of geometric traits obtained by measured and 3D model calculated of peanut plant at seedling stage

Peanut plant	Height (mm)			Width (mm)			Length (mm)			Volume (cm^3^)		
	$M$	$C$	$AP$ (%)	$M$	$C$	$AP$ (%)	$M$	$C$	$AP$ (%)	$M$	$C$	$AP$ (%)
1	96	99	96.88	144	138	95.83	201	214	93.53	2778.62	2923.67	94.78
2	274	273	99.64	220	200	90.91	148	158	93.24	8921.44	8626.80	96.70
3	263	271	96.96	202	175	86.63	176	179	98.30	9350.18	8489.08	90.79
4	232	236	98.28	203	206	98.52	223	201	90.13	10,502.41	9771.82	93.04
5	299	292	97.66	264	250	94.70	215	202	93.95	16,971.24	14,746.00	86.89
6	274	268	97.81	222	210	94.59	176	144	81.82	10,705.73	8104.32	75.70
7	166	173	95.78	212	210	99.06	227	239	94.71	7988.58	8682.87	91.31
8	249	246	98.80	207	216	95.65	163	173	93.87	8401.51	9192.53	90.58
9	338	334	98.82	213	234	90.14	292	291	99.66	21,022.25	22,743.40	91.81
10	201	195	97.01	166	160	96.39	248	223	89.92	8274.77	6957.60	84.08
11	258	254	98.45	259	251	96.91	288	273	94.79	19,244.74	17,404.84	90.44
12	266	269	98.87	192	204	93.75	300	241	80.33	15,321.60	13,225.12	86.32
13	263	261	99.24	288	300	95.83	301	267	88.70	22,798.94	20,906.10	91.70
14	156	160	97.44	151	146	96.69	242	229	94.63	5700.55	5349.44	93.84
15	262	257	98.09	224	235	95.09	178	202	86.52	10,446.46	12,199.79	83.22
16	225	221	98.22	212	218	97.17	215	185	86.05	10,255.50	8912.93	86.91
17	259	256	98.84	202	172	85.15	205	196	95.61	10,725.19	8630.27	80.47
18	283	276	97.53	222	224	99.10	199	229	84.92	12,502.37	14,157.70	86.76
19	281	283	99.29	201	215	93.03	172	154	89.53	9714.73	9370.13	96.45
20	270	263	97.41	226	231	97.79	321	303	94.39	19,587.42	18,408.16	93.98

The $M$ represents the measured values, the $C$ represents calculated values based on the 3D model. The $AP$ represents the accuracy percentage of calculated values, and $AP=(1-|M-C|/M)100\%$

The statistical data of ground- truth and calculated values based on the 3D model for geometric traits of peanut plants at the flowering stage are shown in Table 4. As shown in Table 4, the maximum accuracy of the calculated value of the peanut plant’s height relative to the real value on the ground-truth is 100.00%, and the minimum is 95.59%. The maximum accuracy of the calculated value of the peanut plant’s width relative to the real value on the ground-truth is 98.84%, and the minimum is 88.45%. The maximum accuracy of the calculated value of the peanut plant’s length relative to the real value on the ground-truth is 99.69%, and the minimum is 71.89%. The maximum accuracy of the calculated value of the peanut plant’s space volume relative to the real value on the ground-truth is 99.62%, and the minimum is 73.97%.

**Table 4:** Statistical data of geometric traits obtained by measured and 3D model calculated of peanut plant at flowering stage

Peanut plant	Height (mm)			Width (mm)			Length (mm)			Volume (cm^3^)		
	$M$	$C$	$AP$ (%)	$M$	$C$	$AP$ (%)	$M$	$C$	$AP$ (%)	$M$	$C$	$AP$ (%)
1	156	159	98.08	151	146	96.69	244	234	95.90	5747.66	5432.08	94.51
2	273	273	100.00	309	321	96.12	299	247	82.61	25,222.74	21,645.35	85.82
3	210	219	95.71	225	222	98.67	281	202	71.89	13,277.25	9820.84	73.97
4	297	292	98.32	344	348	98.84	223	232	95.96	22,783.46	23,574.91	96.53
5	281	285	98.58	370	357	96.49	219	236	92.24	22,769.43	24,011.82	94.54
6	319	315	98.75	231	239	96.54	231	212	91.77	17,022.16	15,960.42	93.76
7	268	266	99.25	229	224	97.82	324	287	88.58	19,884.53	17,100.61	86.00
8	270	276	97.78	303	338	88.45	220	210	95.45	17,998.20	19,590.48	91.15
9	361	360	99.72	306	311	98.37	406	359	88.42	44,849.20	40,193.64	89.62
10	247	240	97.17	222	238	92.79	331	289	87.31	18,150.05	16,507.68	90.95
11	331	326	98.49	209	217	96.17	349	303	86.82	24,143.47	21,434.83	88.78
12	299	292	97.66	230	238	96.52	227	205	90.31	15,610.79	14,246.68	91.26
13	311	314	99.04	386	398	96.89	296	278	93.92	35,533.62	34,742.22	97.77
14	204	195	95.59	331	343	96.37	217	220	98.62	14,652.71	14,714.70	99.58
15	336	336	100.00	293	310	94.20	193	188	97.41	19,000.46	19,582.08	96.94
16	315	304	96.51	330	343	96.06	324	325	99.69	33,679.80	33,888.40	99.38
17	252	257	98.02	296	281	94.93	246	219	89.02	18,349.63	15,815.52	86.19
18	337	330	97.92	224	231	96.88	296	292	98.65	22,344.45	22,259.16	99.62
19	314	309	98.41	223	227	98.21	164	174	93.90	11,483.61	12,204.88	93.72
20	321	325	98.75	232	240	96.55	395	368	93.16	29,416.44	28,704.00	97.58

The $M$ represents the measured values, the $C$ represents calculated values based on the 3D model. The $AP$ represents the accuracy percentage of calculated values, and $AP=(1-|M-C|/M)100\%$

**Fig. 7:**
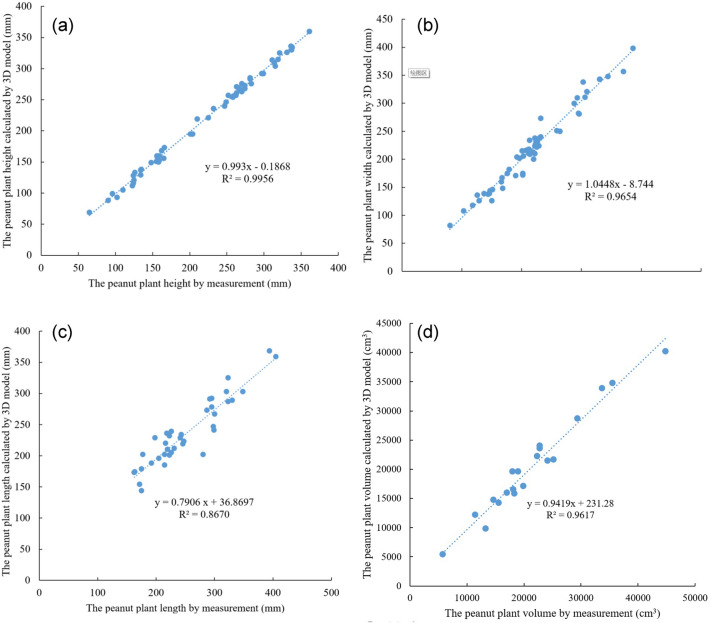
Correlation of peanut plants’ geometric traits between manual ground-truth and model calculations. **a** fitting results of peanut plants’ height, **b** fitting results of peanut plants’ width, **c** fitting results of peanut plants’ length, **d** fitting results of peanut plants’ volume

The average accuracy of peanut plants’ height, width, length, and volume calculated through the 3D model from ground-truth is 97.37%, 95.33%, 90.69%, and 90.28% in all three growth stages, respectively. Figure 7 shows the correlation between the ground-truth measurements and the 3D model calculations for each peanut plant during the whole course of the experiment. It can be seen from Fig. 7, that there is an obvious positive correlation between the manual measured values and model calculated values, and the Goodness of Fit R^2^ for plants’ height, width, length, and volume is 0.9956, 0.9654, 0.8670, and 0.9815, respectively.

Table 5 shows the average accuracy of each evaluation parameter value calculated by the 3D reconstruction model at various growth stages of peanut plants. As shown in Table 5, the average of calculated values of all evaluation parameters is gradually increasing with the growth of peanut plants, and the total average value of the three stages exceeds 90%. The $Acc$, synthetic accuracy, exceeds 92% in all growth stages.

**Table 5:** Accuracy evaluation results of 3D model of peanut at different growth stages

The 3D model evaluation parameters	Values of the sprout stage (%)	Values of the seedling stage (%)	Values of the flowering stage (%)	Average values of all three growth stages (%)
Height	95.88	98.05	98.19	97.37
Width	95.16	94.65	96.18	95.33
Length	89.25	91.23	91.58	90.69
Volume	89.17	89.29	92.38	90.28
$Acc$	92.37	93.30	94.58	93.42

## Discussion

In this section, some interference factors in 3D modeling are analyzed, the influence of parameters setting on statistical filtering results are discussed, and the PSC 3D modeling method proposed in this paper is compared with the ICP-based modeling method in terms of modeling speed.

### Analysis of factors affecting the accuracy of 3D model reconstruction

The depth camera has great application potential for 3D plant reconstruction and the acquisition of phenotypic data. Its advantages include simultaneous acquisition of color and depth information, high accuracy, and low cost of operation [21, 40]. Calibrating the depth camera imaging system and obtaining its precise parameters helps improve the accuracy of the 3D reconstruction model. The acquisition of rotation and translation matrix is the key to generating a complete 3D model of the peanut plant, which reflects the corresponding relationship between the point cloud and the actual spatial location of the target. The depth camera data can be converted to the color camera coordinate system using a rotation and translation matrix. Therefore, in a 3D model reconstruction system with a fixed structure, the calibration of the position of the sensor itself and between the sensors is the premise of building an accurate 3D model.

**Fig. 8:**
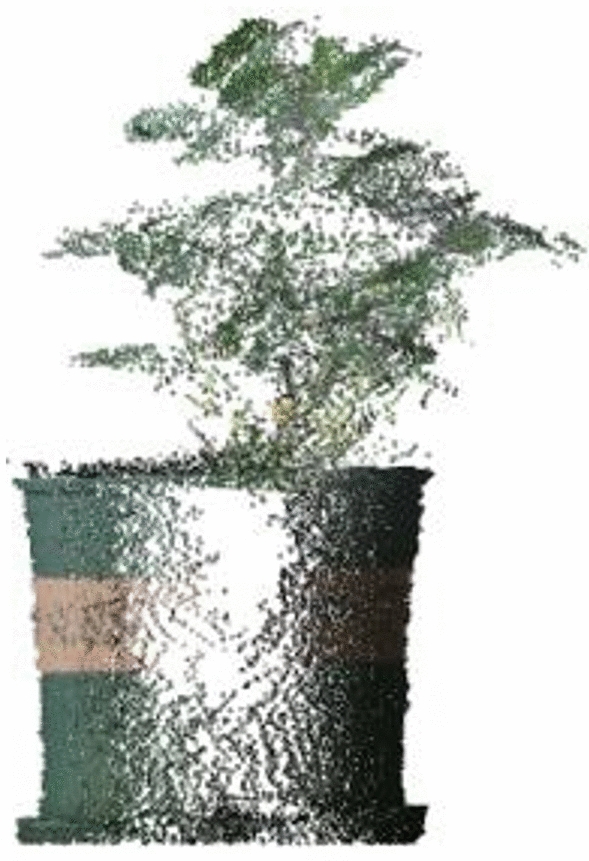
Side view of the 3D reconstruction model with the flower pot

The accuracy of the data in the depth image obtained by Kinect v2 is inconsistent. The error in the center position of the depth image is the smallest, which increases with the distance from the center. The maximum error occurs at the edge of the depth image [63]. This feature leads to the decline of modeling accuracy at the border when reconstructing the 3D model based on the point cloud obtained by Kinect v2, as shown in Fig. 8. As shown in Fig. 8, the 3D model is reconstructed from the point cloud data obtained by Kinect v2 placed on its left and right sides. From the front, the point cloud is absent in the middle part of the flower pot 3D model. There are two possible explanations for this occurrence. First, the middle section represents the edge of the point cloud data acquired by Kinect v2 on the left and right. The inherent characteristics of Kinect v2 lead to the decline of the measurement accuracy of the edge part and the quality of the point cloud of the edge part. The second reason is that a portion of the edge discrete point cloud data is eliminated as noise during the two filtering processes. Although the peanut plant is irregular and the point cloud loss is less than that of the flower pot, the reconstruction accuracy of the 3D model in the length direction is still lower than that of other evaluation parameters, as shown in Table 5.

Additionally, in some studies, some phenotypic parameters calculated by 3D models are compared to manually measured values to ensure that reconstructed models are accurately evaluated [26, 53]. However, there are certain irregularities and uncertainties in plant growth. There is a risk of error increase regardless of whether it is measured manually or calculated using a model. Moreover, the plant is a non-rigid structure susceptible to external interference, such as wind, resulting in sway, which affects the evaluation of modeling accuracy. The result is more objective if the 3D model accuracy is evaluated with the height, width, length, and volume of the plant. Thus, by evaluating the accuracy of 3D model reconstruction using multiple phenotype parameters, we can avoid the uncertainty introduced by a single evaluation index of plant.

### Influence of parameters setting on the statistical filtering effect

Selecting the number of adjacent points m and the effective point cloud range coefficient k directly affects the filtering effect in statistical filtering. When $k$ equals 1.0, Table 6 illustrates the effect of various $m$ values on the number of filtered point clouds and 3D modeling accuracy. The data in Table 6 is the average of 60 three-dimension models constructed by all 20 peanut plants in three growth stages. As illustrated in Table 6, the number of filtered point clouds increases with the increase of the m value. On the assumption that the three-dimensional structure of the peanut plant is not harmed, the more point clouds filtered out, the more effective the filtering effect and the faster the post-processing speed. The highest accuracy of the 3D model is 92.39%, which occurs when the $m$ value is 35. At this time, the number of filtered point clouds is at a medium level, so the $m$ value of 35 is appropriate.

**Table 6:** Comparison of filtering effect when $k$ value is 1.0 and m takes different values

Number of point clouds before filtering	$m$ value	Number of filtered point clouds	Number of remaining point clouds	3D model accuracy (%)
9675	65	774	8901	92.14
9675	60	767	8908	92.02
9675	55	762	8913	92.35
9675	50	757	8918	92.30
9675	45	750	8925	92.35
9675	40	742	8933	92.29
9675	35	733	8942	92.39
9675	30	728	8947	92.03
9675	25	725	8950	91.99
9675	20	716	8959	92.11
9675	15	710	8965	92.32

Table 7 shows the results of the point cloud filtering when the $m$ value is 35 and the $k$ value is varied. The data in Table 7 is the average of 60 three-dimension models constructed by all 20 peanut plants in three growth stages. It can be seen from Table 7 that with the larger value of $k$, the filtering effect on outliers decreases. With the smaller value of $k$, the filtering effect is strengthened, and the number of point clouds remaining after filtering decreases. The highest accuracy of the 3D model is 92.39%, which occurs when the $k$ value is 1.0. At this time, the number of filtered point clouds is at a medium level, so the $k$ value of 1.0 is appropriate. The statistical filtering parameters for peanut plants are determined after the test and analysis, which is $k$ = 1.0 and $m$ = 35.

**Table 7:** Comparison of filtering effect when $m$ value is 35 and $k$ has different values

Number of point clouds before filtering	$k$ value	Number of filtered point clouds	Number of remaining point clouds	3D model accuracy (%)
9675	1.4	461	9214	91.81
9675	1.3	515	9160	91.89
9675	1.2	580	9095	92.01
9675	1.1	652	9023	92.11
9675	1.0	733	8942	92.39
9675	0.9	835	8840	92.27
9675	0.8	951	8724	92.34
9675	0.7	1088	8587	92.32
9675	0.6	1261	8414	92.24
9675	0.5	1461	8214	92.19

### Analysis of 3D modeling speed

The accuracy of the PSC 3D model reconstruction method proposed in this paper has been evaluated in “Accuracy evaluation result of 3D Model” section. In addition to the accuracy, the modeling speed is also an important indicator to assess the modeling method. An experiment comparing with the iterative closest point (ICP) algorithm was carried out to verify the modeling speed of the PSC method. Currently, the ICP algorithm is the most widely used method for reconstructing 3D point clouds. The ICP algorithm locates the same target point in two different clouds, calculates their position relationship, and then splices the point cloud through this relationship to reconstruct the 3D model. The ICP algorithm's primary objective is to determine the geometric relationship between corresponding points in two-point clouds. It cannot be applied if there is no corresponding point in two-point clouds. A comparative test is used to compare the proposed method's modeling speed to that of the ICP algorithm. One peanut plant was randomly selected and placed on a rotating table for the test. The Kinect v2 was used to collect RGB and depth data once every ten degrees of rotation, and the data were numbered from Pre1 to Pre36 for a total of 36 times. The three pieces of point clouds obtained from the positions with an interval of 120° as a group was used for the modeling by the ICP algorithm. For example, Group1-ICP includes point clouds obtained from shooting angles of 0° (Per1), 120° (Per 13), and 240° (Per 25), respectively. Additionally, the two pieces of point clouds obtained from two positions separated by 180° as a group was used for the PSC modeling. For example, Group1-PSC includes point clouds obtained from shooting angles of 0° (Per1) and 180° (Pre19). A total of 36 pieces of point clouds were combined into 12 groups using this method, and each group was different from the others. The comparative tests' statistical results are summarized in Table 8.

**Table 8:** The statistical results of the contrast test of 3D reconstruction of peanut plant through the ICP and PSC algorithm, respectively

Contrast test	Modeling time		Model accuracy	
	ICP	PSC	ICP (%)	PSC (%)
Group1	5.740 s	2.099 s	90.54	94.54
Group 2	5.953 s	2.157 s	89.87	91.89
Group 3	4.889 s	2.136 s	90.54	92.57
Group 4	5.200 s	2.081 s	95.27	94.22
Group 5	5.434 s	2.042 s	92.57	92.64
Group 6	5.157 s	2.198 s	95.62	91.92
Group 7	4.618 s	2.032 s	96.62	92.53
Group 8	5.618 s	2.129 s	96.92	92.87
Group 9	5.635 s	2.355 s	97.97	93.58
Group 10	5.579 s	2.069 s	98.65	94.58
Group 11	5.604 s	2.278 s	97.97	92.64
Group 12	5.716 s	2.088 s	95.32	95.60
Mean value	5.429 s	2.139 s	94.82	93.30

As shown in Table 8, the reconstruction time for the 3D model using the ICP algorithm ranges between 4.618 s and 5.953 s, with an average of 5.429 s. Similar results were also obtained in the study of Yuan et al. [64], in which four RGB-D cameras were used to collect data, and the foot 3D model reconstruction takes approximately 5 s by the ICP algorithm. This shows that there is no significant difference in the time cost of scanning the target from three or four angles and reconstructing the 3D model according to the ICP algorithm. ICP algorithm requires that point clouds from different views must have overlapping parts, which is similar to SV and SfM. The more overlap of the point clouds, the higher accuracy of the 3D reconstruction model, but the more time spent for modeling. Hu et al. [49] scanned the leafy vegetables from 18 views and used ICP algorithm to model them. It spent at least 3.73 min to process the data of a vegetable.

The ICP algorithm is powerless if there is no overlap in the point cloud. The PSC algorithm proposed in this paper can effectively solve this problem, and the PSC algorithm reconstructs the 3D model in 2.032 s to 2.355 s with an average of 2.139 s for the peanut plants. The model's accuracy obtained by the ICP algorithm ranges from 89.87 to 98.65%, with an average of 94.82%. The model’s accuracy obtained using the PSC method ranges from 91.89 to 95.60%, with an average of 93.30%. Compared with other plant 3D modeling methods, the modeling accuracy of the method proposed in this paper has obvious advantages [17, 21]. The accuracy of the PSC algorithm for 3D model reconstruction is 1.52 percent lower than that of the ICP algorithm, but the time consumed for the 3D reconstruction only is 39.4% of the ICP algorithm. The PSC algorithm can reconstruct a 3D model close to the accuracy of the ICP algorithm at a speed of 2.54 times, which demonstrates the performance ratio advantages of the PSC algorithm.

## Conclusions

In this paper, a 3D model reconstruction method of the peanut plant based on Kinect v2 was designed. Two Kinect v2s were used to generate a 3D model of the peanut plant through data filtering and coordinate transformation. The experiment was conducted at various stages of peanut growth, and the 3D models were evaluated using the synthetic accuracy based on the plant height, width, length, and volume of the peanut, respectively. The experimental results indicate that the peanut plant 3D reconstruction model's accuracy is 92.37%, 93.30%, and 94.58% at the sprout stage, seedling stage, and flowering stage respectively, and 93.42% for the growth stages. Compared to the ICP method, the proposed method is 2.54 times faster with closed accuracy. The reconstruction method for the 3D model of the peanut plant described in this paper is capable of rapidly and effectively establishing a 3D model of the peanut plant while also meeting the modeling requirements for other species' breeding processes. In subsequent research, we will attempt to reconstruct the three-dimensional model of the plant of multiple peanuts simultaneously.

## Data Availability

All data generated or analyzed during this study are available from the corresponding author on reasonable request.
